# Mightier Than the Pen

**Published:** 1875-10

**Authors:** 


### MIGHTIER THAN THE PEN.

If we were to announce that we employed a system of penmanship, in our correspondence and in our editorial work, which, while producing writing not only as plain as print, but actually in printed characters, we should astonish our readers. If we were to add to this statement, that we could write in this legible manner much faster than any body else could in the ordinary way—twice as fast, at least—their astonishment would be greatly increased.

Well, now, we know of a plan by which all this can be done. There is on exhibition at the Fair of the American Institute, a little machine with which any person can write much faster than in the old way, after a few hours' experience. The writing is, as we have intimated, as perfect as printing, because, instead of making scratches as the pen does, it gives an impression from the face of a type for each letter or character, and this secures perfect legibility whether the individual possesses the writing facility or otherwise.

The machine of which we speak is called a Type-Writer, because it writes with types. It is a neat little instrument, before which the writer sits and drums away as he would at a piano. A series of little knobs are arranged before him; and, as these are depressed, the letter or character exhibited on the knob is reproduced on the paper. The operation is very rapidly performed,—an expert writer of either sex accomplishing about 80 words a minute without difficulty.

The mechanical labor of ordinary pen and pencil writing, is serious and exhausting. If long continued the influence upon the system is sure to be injurious. The attitude of the writer is bad, for his head must be lowered, and the tendency is for his body to be bent. The lungs are contracted nine times out of ten, by the position assumed, and the stomach and bowels are not allowed freedom of action, in consequence of the stooping posture into which the person surely falls, after long-continued work at the desk. The right hand, too, loses its power after a time, from overwork of a sort which does not increase the strength, because the circulation is impeded instead of quickened, and "pen-paralysis" supervenes. Nobody knows what hard, exhausting labor writing is, except those who have worked at it day in and day out. The Type-Writer cures all this, and converts an enervating duty into a very fascinating pastime. Take a reporter who has been reporting in court all day, and who is expected to write out his shorthand notes at night, and let him sit down at one of these little machines and drum out his clear, beautiful manuscript, in printed form, ready for any purpose, without the intervention of the type-setter, and he will tell you that he has made an enormous saving in muscle, and time, and labor, and money.

The machine is simplicity itself; a child of intelligence can soon learn to operate it; and ladies, with their nimble fingers, quick motions, and natural aptness, become in a day or two rapid writers. It is a wonderful educator, too, because it teaches spelling, and composition, and punctuation, by exhibiting one's errors in these respects, so clearly as to make their correction an absolute necessity.

We were trying to think what class of people would find this instrument most useful, and were forced to conclude that there are very few who would not be better off for possessing it. Perhaps the good lady who wrote us the other day, and began by saying that the letter was the first one she had written since postage-stamps came into use, would not find the Type-Writer particularly useful, though we are confident that if she had owned one all these years she would have written a good many letters, because the labor would have been greatly simplified, and her hand-writing—cramped and bad, of course—would have been as good as printing, so that she would not have felt ashamed to have it seen. The mass of intelligent people would find this instrument a great convenience, as well as a real saving in time and money. But all who write much, who correspond largely, who prepare matter for the press, who copy documents, in whose hand the pen or the pencil is found during some hours of each day, all these will find a very great advantage in the use of this machine. It will not wholly supersede the use of the pen, but it is destined to take its place with all whose lives are given up to the laborious duty of wielding that potent weapon. It is mightier than the pen, because it works with greater speed, and involves less wear and tear to the human frame. Messrs. Densmore, Yost, and Co., No. 707 Broadway, are the General Agents, and will send descriptive illustrated circulars on application.

